# Hippocampal neurons respond to brain activity with functional hypoxia

**DOI:** 10.1038/s41380-020-00988-w

**Published:** 2021-02-09

**Authors:** Umer Javed Butt, Agnes A. Steixner-Kumar, Constanze Depp, Ting Sun, Imam Hassouna, Liane Wüstefeld, Sahab Arinrad, Matthias R. Zillmann, Nadine Schopf, Laura Fernandez Garcia-Agudo, Leonie Mohrmann, Ulli Bode, Anja Ronnenberg, Martin Hindermann, Sandra Goebbels, Stefan Bonn, Dörthe M. Katschinski, Kamilla W. Miskowiak, Klaus-Armin Nave, Hannelore Ehrenreich

**Affiliations:** 1 Clinical Neuroscience, Max Planck Institute of Experimental Medicine, Göttingen, Germany; 2 Department of Neurogenetics, Max Planck Institute of Experimental Medicine, Göttingen, Germany; 3 Institute of Medical Systems Biology, Center for Molecular Neurobiology, University Clinic Hamburg-Eppendorf, Hamburg, Germany; 4 Institute for Cardiovascular Physiology, University Medical Center Göttingen, Georg-August-University, Göttingen, Germany; 5 Psychiatric Centre Copenhagen, University Hospital, Rigshospitalet, Copenhagen, Denmark

**Keywords:** Neuroscience, Molecular biology

## Abstract

Physical activity and cognitive challenge are established non-invasive methods to induce comprehensive brain activation and thereby improve global brain function including mood and emotional well-being in healthy subjects and in patients. However, the mechanisms underlying this experimental and clinical observation and broadly exploited therapeutic tool are still widely obscure. Here we show in the behaving brain that physiological (endogenous) hypoxia is likely a respective lead mechanism, regulating hippocampal plasticity via adaptive gene expression. A refined transgenic approach in mice, utilizing the oxygen-dependent degradation (ODD) domain of HIF-1α fused to CreERT2 recombinase, allows us to demonstrate hypoxic cells in the performing brain under normoxia and motor-cognitive challenge, and spatially map them by light-sheet microscopy, all in comparison to inspiratory hypoxia as strong positive control. We report that a complex motor-cognitive challenge causes hypoxia across essentially all brain areas, with hypoxic neurons particularly abundant in the hippocampus. These data suggest an intriguing model of neuroplasticity, in which a specific task-associated neuronal activity triggers mild hypoxia as a local neuron-specific as well as a brain-wide response, comprising indirectly activated neurons and non-neuronal cells.

## Introduction

Hypoxia is the term for reduced oxygen levels in cells or tissues, relative to their ‘normal’ content. In former times interpreted as principally pathological, for instance upon cardiac arrest, hypoxia is increasingly recognized as physiological driving force of early neurodevelopment including angiogenesis, hematopoiesis, and tissue regeneration. Known cellular environments experiencing hypoxia include developing embryos, stem cell niches, the renal papilla, inflammatory tissue, or the inner mass of tumours [1–10]. A specific transcriptional programme, induced by hypoxia, allows cells to adapt to lower oxygen levels and/or to limited metabolic support [11]. The transcription is partly independent of [10, 12] and partly controlled by hypoxia-inducible factors (HIF), binding to hypoxia-responsive elements to modulate expression of a myriad of genes, some of which are potent growth factors like vascular endothelial growth factor (VEGF) or erythropoietin (EPO) [10, 13–20].

On one hand, the tight association of neuronal activity with oxygen availability is the basis of functional magnetic resonance imaging (fMRI), which works by detecting the level of oxygen in blood throughout the brain. Changes in oxygenation and hemodynamics generate a fast surrogate signal of brain activity based on structural and functional neurovascular coupling [21–23].

On the other hand, extensive physical activity as well as cognitive challenge lead to widespread brain activation, and are ultimately associated with improved global brain function including mood and emotional well-being in health and disease [24, 25]. Neurologists and psychiatrists encourage their patients to improve functions by practicing, following the old rule *‘use-it-or-lose-it’*. For example, hippocampal volume increases following exercise in both healthy and schizophrenic subjects, and this plastic response correlates with improvement in test scores for short-term memory [26]. Despite these well-established observations, the underlying mechanisms have remained widely obscure.

In several clinical trials targeting different neuropsychiatric diseases, we showed over the last 2 decades that high-dose recombinant human EPO consistently improved cognition and reduced grey matter loss (e.g. [27–30]). Subsequently focusing on preclinical EPO studies for deeper mechanistic insight, we discovered that challenging cognitive tasks apparently induce transient neuronal hypoxia which triggers neuronal EPO expression. Endogenous EPO in turn enhances cognition via augmenting dendritic spine formation and increasing numbers of pyramidal neurons [31, 32]. In this context, we coined the term *‘brain EPO circle’*, and reported an increase in hypoxia-labelled neurons after complex running wheel (CRW) exposure together with an amplified expression of EPO in pyramidal CA1 neurons [32].

Taking all this information together, we designed the present study, hypothesizing that for physiological postnatal and adult adaptation processes in the brain, hypoxia may be a crucial mediator of major general relevance. We show here that complex motor-cognitive activity leads to ‘functional hypoxia’ as a local, neuronal network-specific, as well as a brain-wide response, encompassing indirectly activated neurons and—to a lesser degree—non-neuronal cells. This activity-induced hypoxia regulates adaptive gene expression and fosters neuroplasticity.

## Materials and methods

**Important note:** All experiments, including cell counting, were performed by investigators unaware of group assignment and treatments (‘fully blinded’).

### Generation of CAG-CreERT2-ODD transgenic mice

**Fig. 1:**
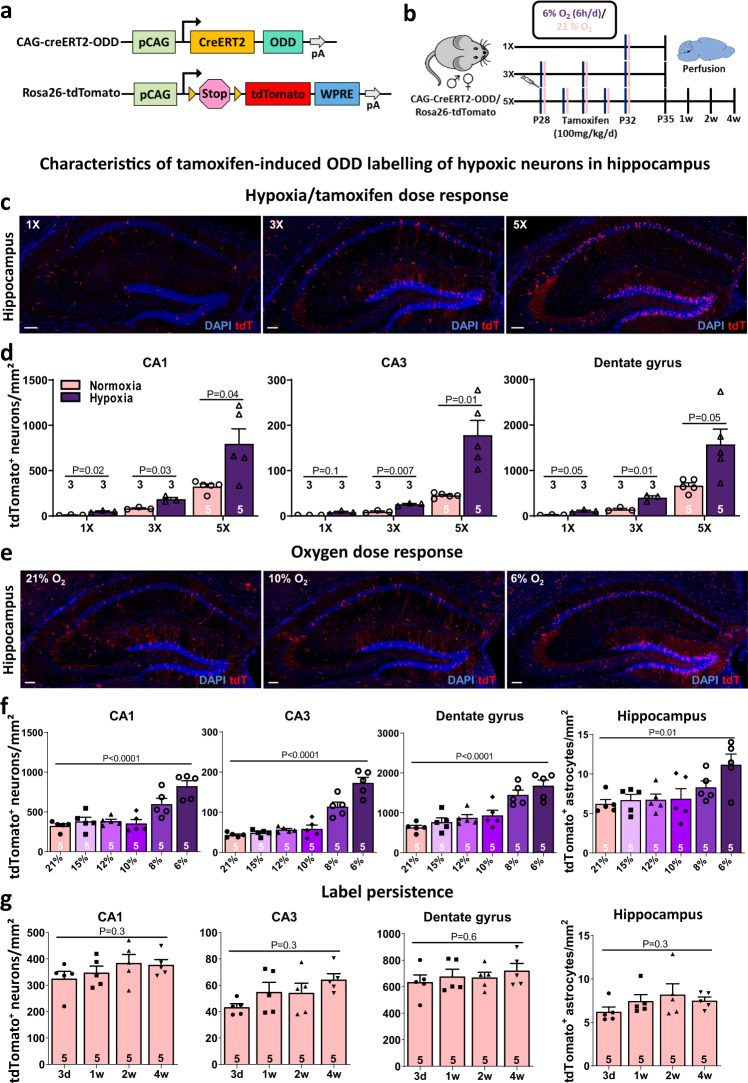
Generation and characterization of CAG-CreERT2-ODD::R26R-tdTomato mice as model to study hypoxic cells in the brain. **a** CAG-CreERT2-ODD and CAG-Rosa26R-tdTomato constructs. **b** Experimental outline for quantification of hypoxic cells in hippocampal regions CA1, CA3, and dentate gyrus under variable conditions, as displayed in **(c–g**). After the last day of treatment (P32), mice were sacrificed, perfused and brains harvested for histological analysis at the time points indicated. **c** Illustrative images of hippocampus from 1×, 3× and 5× hypoxia/tamoxifen-treated mice, showing tdTomato+ (red) hypoxic cells; DAPI (blue) as nuclear stain. **d** Quantitative results (based on counting of tdT+NeuN+ neurons) of the hypoxia/tamoxifen titration scheme, combining 1×, 3× or 5× placement of mice right after tamoxifen injection (100 mg/kg/d i.p.) for 6 h into a cage with hypoxia exposure (6%O_2_) versus a cage with continued normoxia (21%O_2_). **e** Illustrative images (5× tamoxifen), showing tdTomato+ (red) hypoxic cells and DAPI (blue) as nuclear stain and **f** quantification of the dose-response of hypoxic neurons (based on tdT+NeuN+ cells) and astrocytes (based on tdT+S100β+ cells) upon decreasing inspiratory O_2_ concentration (5× tamoxifen; 21%O_2_, 15%O_2_, 12%O_2_, 10%O_2_, 8%O_2_ and 6%O_2_). **g** Tracking of tamoxifen-induced tdTomato+ hypoxic cells over the course of 4 weeks after treatment cessation under normoxic conditions (5× tamoxifen) reveals essentially label persistence; 2-tailed Welch’s *t*-test and 1-way ANOVA were used for statistical analyses; error bars indicate SEM; scale bar represents 100 µm.

The p-CAG vector, kindly provided by Hesham A. Sadek [33], was slightly modified (Fig. 1a). In brief, the vector contains the oxygen-dependent-degradation domain (ODD) of HIF-1α, fused with a tamoxifen-inducible cre-recombinase, driven by a ubiquitous CAG promoter. Under normoxic conditions, the ODD is hydroxylated by prolyl-hydroxylases, which tag HIF-1α upon binding of Von Hippel-Lindau (VHL) protein for proteasomal degradation. Hypoxic conditions lead to inactivation of these enzymes and thus stabilization of HIF-1α, allowing it to accumulate in the nucleus and activate transcription of its target genes. Analogously, under hypoxic conditions the ODD-cre-recombinase fusion construct is stabilized and—in the presence of tamoxifen—translocates to the nucleus to trigger tdTomato expression, which leads to permanent labelling of cells that were transiently exposed to hypoxia [33]. The vector was cloned and bacterial backbone (ampicillin sequence) removed by DraI and SpeI (New England Biolabs, MA, USA) restriction enzymes. The linearized vector for pro-nuclear microinjection was purified by QIAquick Gel Extraction kit (Qiagen, Venlo, Netherlands) and introduced into fertilized eggs for generation of CAG-CreERT2-ODD transgenic mice. Transgenic founder mice and the next generations were viable and breeding normally.

### Mouse genotyping

Founder mice from the litters were screened by PCR amplification of genomic DNA for the transgene using following primer pair: forward 5′-GCTGAAGACACAGAAGCAAA-3′ and reverse 5′-GTGGGTAGGAGATGGAGATG-3′. Mice carrying the transgene were maintained on C57BL6/N (Charles River, MA, USA) background. F1 litters were bred to Rosa26R-tdTomato reporter mice [34]. For analysis of tdTomato transgene, primer1 5′-TCAATGGGCGGGGGTCGTT-3′, primer2 5′-CTCTGCTGCCTCCTGGCTTCT-3′ and primer3 5′-CGAGGCGGATCACAAGCAATA-3′ were used. CAG-CreERT2-ODD::R26R-tdTomato F2 litters were selected for histological analysis.

### Experimental procedures

All experiments were approved by and conducted in accordance with the regulations of the local Animal Care and Use Committee (Niedersächsisches Landesamt für Verbraucherschutz und Lebensmittelsicherheit, LAVES). CAG-CreERT2-ODD::R26R-tdTomato mice were used at the age of 4 and 24 weeks in all experiments. Mice were single housed in standard plastic cages, starting 2–3 days before the respective experiments, and maintained in temperature-controlled environment (21 ± 2 °C) on 12 h light–dark cycle with food and water available *ad libitum*. Mice of each gender were randomly allocated to experimental and control groups.

#### **Tamoxifen injections**

Tamoxifen stock solution was prepared by mixing 300 mg tamoxifen (Sigma, Darmstadt, Germany) in 30 ml of corn oil (Sigma) and stored at 4 °C; 100 mg/kg tamoxifen was injected intraperitoneally (i.p.) after preheating and sonication for 20 min. To control for non-induced expression of the transgene (‘leakiness’), corn oil was injected.

#### Hypoxia exposure

A hypoxia chamber (60 cm × 38 cm × 20 cm) was designed in cooperation with Coy Laboratory Products (Michigan, USA). The hypoxia chamber is equipped with an oxygen sensor, oxygen controller and a ceiling fan for constant air circulation. In hypoxia experiments (Fig. 1b), animals were given a single dose of tamoxifen (100 mg/kg) before being placed in the hypoxia chamber for 6 h at 6%O_2_. This procedure was repeated at identical time (10.30–16.30 h) for 5 consecutive days if not stated otherwise. O_2_ was dropped from 21 to 6% over the course of 30 min, kept at 6% for 6 h and brought back up to 21% upon cessation of the experiment. Mice included in the normoxia group and oil-only control mice were handled identically but stayed at 21%O_2_. For histological studies mice were perfused 3 days after the 5-day experiment (day 8) and brains dissected for further analysis. For single-cell RNAseq analysis, mice were sacrificed immediately after the last hypoxia/normoxia control session on day 5 without perfusion.

#### Running wheel experiment

CRW (TSE Systems, Bad Homburg, Germany) are characterized by randomized missing bars as previously described [35, 36] and running activity is computer-controlled via Phenomaster software (TSE Systems, Germany). Running-naïve mice, aged 4 or 24 weeks, were allowed to freely use CRW for 5 whole days. Tamoxifen injections were given daily during the light cycle, along with refill of fresh food and water. Non-running mice were excluded from the experiment (5/24 all together). Control mice were housed without CRW in standard cages. After day 5, CRW mice were returned to standard cages for 3 days before perfusion (day 8).

### Tissue preparation and histology

Mice were anesthetized with Avertin (Tribromoethanol, Sigma-Aldrich, St Louis, MN, USA, 0.276 mg g^–1^) and perfused with 0.9% saline and pre-chilled 4% paraformaldehyde (PFA; Sigma, Missouri, United States), followed by brain isolation. All tissues were post-fixed in 4% formaldehyde at 4 °C for 24 h and later cryoprotected in 30% sucrose prepared in phosphate buffer saline (PBS) for 48 h at 4 °C. Brains were further covered in cryoprotectant (O.C.T.^TM^ Tissue-Tek, Sakura, Netherlands) and kept until use at −80 °C. Whole mouse brains were cut into 30 μm thick coronal floating sections using a cryostat (Leica, Wetzlar, Germany) and stored in a cryoprotective solution (25% ethylene glycol and 25% glycerol in PBS) at 4 °C until further use.

#### Immunofluorescence staining

For immunofluorescence analysis, 5 sections (30 µm) from each mouse were washed and blocked for 1 h in 5% horse serum diluted in PBS with 0.5% Triton X-100 at room temperature (RT). Primary antibodies were diluted in 3% horse serum PBS/0.3% Triton X-100 and sections incubated for 48 h at 4 °C, followed by washing and incubation with respective secondary antibody for 2 h at RT. For nuclear counterstaining, 4′,6-diamidino-2-phenylindole (DAPI, Sigma, Missouri, United States) was used. The sections were then mounted on SuperFrostPlus Slides (Thermo Fisher, Germany) with Aqua-Polymount (Polysciences, Inc USA). For direct visualization of tdTomato, sections were only stained with DAPI and investigated by confocal microscope Leica TCS SP5-II. Primary antibodies used were anti-NeuN (MAB377, 1:1000, Mouse Millipore, Darmstadt, Germany), anti-IBA1 (234006, 1:1000, chicken; SYSY, Göttingen, Germany) and anti-Olig2 (AB9610, 1:1000, rabbit, Millipore), anti-alanyl aminopeptidase membrane (CD13)-AF647 (564352, rat, 1:200, 564352; BD Biosciences, San Jose, CA, USA), Lectin (DL-1174, 1:500, DyLight^®^ 488 Lycopersicon Esculentum (Tomato), anti-S100β (287004/1-4, guinea pig, 1:500, SYSY). Secondary antibodies used were Alexa 488 anti-guinea pig (123588, 1:500, Invitrogen), Alexa 647 anti-chicken (121361, 1:500, Jackson ImmunoResearch, Cambridgeshire, UK), Alexa 647 anti-mouse (1757130), Alexa 647 anti-rabbit (1693297) and Alexa 647 anti-rat (A-21247, 1:500; Thermo Fisher).

#### Confocal imaging and quantification

For quantification of NeuN+tdTomato+ co-labelled cells, serial coronal sections from the dorsal part of hippocampus were taken (coordinates from Bregma: −1.34 to −2.54 mm posterior). Stained sections (30 µm) were imaged using a Leica confocal microscope TCS SP5 System equipped with a 20× objective (NA = 0.70). Neurons (NeuN+) from CA1, CA3 and DG were counted on both sides of hippocampus by Fiji software (https://imagej.net/Fiji). NeuN+tdTomato+ cells were considered as neurons having experienced hypoxia. Astrocytes (S100β+tdTomato+) and endothelial cells (Lectin+tdTomato+) were counted bilaterally in whole hippocampus (5 sections per animal). For oligodendrocyte quantification, Olig2+tdTomato+ cells were counted in serial coronal sections of the rostral corpus callosum (both sides). Illustrative images were analyzed and processed with Imaris 9.1.0 (www.bitplane.com).

### Light-sheet microscopy (LSM)

#### Whole mount tissue staining and clearing

To visualize tdTomato+ cells in the entire brain we performed LSM in combination with whole mount immune labelling and tissue clearing. Animals were deeply anesthetized and transcardially perfused using PBS and 4%PFA/PBS, respectively. Brains were removed, post-fixed in 4%PFA overnight and stored in PBS until further use. Brain hemispheres were processed for immune labelling and tissue clearing following a slightly modified iDISCO protocol [37]. Samples were dehydrated with a methanol/PBS series (50%, 80%, 100%, and 2 × 100%, 1 h, RT) followed by overnight bleaching and permeabilization in a mixture of 5% H_2_O_2_/20% dimethyl sulfoxide (DMSO) in methanol at 4 °C. Samples were retrieved and washed further with methanol at 4 °C for 30 min and −20 °C for 3 h prior to incubation in 20% DMSO in methanol at RT for 2 h. Samples were then rehydrated using a descending methanol/PBS series (80%, 50%, PBS, 1 h each, RT) and further washed with in PBS/0.2% Triton X-100 for 2 h. The samples were then incubated overnight in 0.2% Triton X-100, 20% DMSO, and 0.3 M glycine in PBS at 37 °C and blocked using PBS containing 6% goat serum, 10% DMSO and 0.2% Triton X-100 for 2 days at 37 °C. Samples were retrieved, washed twice in PBS containing 0.2% Tween(r)20 and 10 µg/ml heparin (PTwH) at RT for 1 h and incubated with primary antibody solution (anti-RFP; Rockland # 600-401-379; 1:250 in PTwH/5%DMSO/3% goat serum) for 14 days at 37 °C. After several washes during the day and an additional overnight wash in PTwH samples were incubated with secondary antibody solution (goat anti-rabbit Alexa555; Thermo Fisher Scientific # A-21428; 1:500 in PTwH/3% goat serum) for 7 days at 37 °C. Prior to clearing, the samples were again washed in PTwH (several solution changes during the day) followed by an additional overnight wash. Tissue was dehydrated using an ascending series of Methanol/PBS (20%, 40%, 60%, 80%, 2 × 100% 1 h, RT) followed by overnight incubation in a mixture of 33% dichloromethan (DCM) and 66% methanol at RT. Samples were further delipidated by incubation in 100% DCM for 40 min and transferred to pure ethyl cinnamate (Eci; Sigma-Aldrich #112372) as clearing agent. Tissues became transparent after 30 min in Eci and were stored at RT until imaging.

#### LSM and 3D analysis/visualization

LSM was performed using a LaVision Ultramicroscope II equipped with a 2× objective, corrected dipping cap and zoom body. Samples were mounted onto the sample holder with the medial surface of the brain hemisphere facing down in order to acquire sagittal images. The holder was placed into the imaging chamber filled with Eci. Images were acquired in mosaic acquisition mode with the following specifications: 5 µm sheet thickness; 20% sheet width; 2× zoom; 4 × 5 tiling; 4 µm z-step size; dual site sheet illumination; 50 ms camera exposure time; 1000px × 1600px field of view. Red fluorescence was recorded using 561 nm laser excitation (5–10%) and 585/40 emission filters. Images were loaded into Vision4D 3.0 (Arivis) and stitched using the tile sorter setup. Hippocampus and cortex regions of interests (ROIs) where manually annotated according to the sagittal Allen mouse brain atlas [38]. For this, hippocampus and cortex ROIs were traced manually in a few planes in 2D from which the 3D ROI was extrapolated automatically. Cortex and hippocampus annotations were then cropped with a medial cut-off of ~0.4 mm and a lateral cut-off of ~4.4 mm (corresponding to the lateral end of the hippocampal formation). Cortex ROIs spanned the dorsal parts of the cortex as defined by anatomical landmarks. Next, tdTomato+ cells per ROI were identified using the blob finder algorithm in Vision4D. Noise was removed by deleting objects with voxel sizes <10 from the object table. Objects were then critically reviewed, and any additional noise was manually removed from the dataset. The number of tdTomato+ cells per ROI was extracted from the object table and plotted using GraphPad (Prism). For visualization purposes, representative single planes of the 3D datasets displayed in inverted grayscale were extracted. To clearly visualize the distribution of tdTomato+ cells in the 3D hippocampus, the datasets were cropped along the respective hippocampus ROI, the grayscale 3D renderings were inverted, and a high resolution snapshot of the 3D ROIs was taken. Blobfinder identified objects were further displayed as simplified spheres using the centroid display mode of objects in Vision4D.

### Single-cell RNA sequencing

#### Isolation and preparation of whole hippocampal cells

CAG-CreERT2-ODD::R26R-tdTomato mice, 4 weeks old, received tamoxifen (100 mg/kg/d) daily for 5 consecutive days before being exposed to either inspiratory hypoxia (6%O_2_) or normoxia (21%O_2_) for 6 h each. Mice were handled identically and sacrificed on day 5 immediately after the last hypoxia/normoxia session. Hippocampi were dissected in Earle’s Balanced Salt Solution (EBSS1) and later digested with a working solution of Papain/DNaseI prepared in EBSS1, according to the manufacturer’s instructions (Worthington Biochemical Corp). All steps were carried out on ice except the Papain/DNaseI dissociation. The samples were then incubated at 37 °C for 40 min with constant shaking in water bath and switched to 5%CO_2_ every 10 min. Papain/DNaseI was removed and samples were further diluted in 5 ml EBSS2. Dissociated tissues were manually triturated (avoid air bubbles), followed by centrifugation at 900 rpm for 10 min at 4 °C. Supernatant was discarded and the cell pellet gently resuspended in 200 µl of EBSS2 until a smooth and creamy suspension was obtained. Dissociated cells were washed frequently with DMEM/F12 (Sigma) without phenol-red containing 3% foetal bovine serum (FBS; Life Technologies) and centrifuged at 900 rpm for 10 min at 4 °C. Cell pellet was resuspended in 1 ml of DMEM/F12 and filtered through a 70 µm strainer cap (Corning) smoothly to harvest a clear cell suspension. Cell viability and yield was calculated by mixing an equal volume of acridine orange and propidium iodide and checked at the cell counter (Nexcelom Bioscience, MA, USA). Cell suspension (1 ml) was diluted with 3 ml PBS containing 0.04% BSA and centrifuged. Supernatant was removed and cell pellet resuspended in 400 µl PBS containing 0.04% BSA. Cells were fixed by adding 1.6 ml methanol dropwise and kept at −20 °C for 30 min and stored at −80 °C for further use.

#### Cell rehydration, single-cell library preparation and RNA sequencing (scRNA-seq)

The Methanol fixed cell suspensions were stored at −80 °C for less than 7 days before processing. For each sample, ~1.5 million cells were taken for rehydration and downstream scRNA-seq experiment. Cells were first placed on ice and equilibrated to 4 °C. After equilibration, cells were washed 2 times by 1 ml pre-chilled rehydration buffer (1.0% BSA, 0.5U/µl RNAse inhibitor in 1× Dulbecco’s Phosphate-Buffered Saline, DPBS). Centrifugation was done at 3000 rcf for 10 min at 4 °C. Finally, cells were resuspended in pre-chilled 1× DPBS with 0.04% BSA and 0.5U/µl RNase inhibitor to achieve a concentration of 1000 cells/µl. Rehydrated cells were immediately used for the scRNA-seq using the 10× genomics chromium single-cell gene expression platform. Around 35,000 cells from each sample were loaded on 1 well of 10× Single Cell A chip, where each single cell was lysed and its transcriptome combined with a single barcoded gel bead in an oil droplet. Barcode cDNA libraries were then prepared using Chromium Single Cell 3′ Library and Gel-beads kit v2 according to the manufacturer’s instructions. Library quality was checked using Agilent High Sensitivity DNA chip on Agilent 2100 Bioanalyzer. High quality libraries were sequenced on Illumina HiSeq 4000 sequencer with an average depth of 200,000 reads per cell.

#### Alignment and initial processing of sequencing data

CellRanger v2.2.0 software was used to align sequence reads to the customized mouse mm10 reference genome to which the ODD and tdTomato sequences were added. A filtered gene expression matrix was generated by CellRanger and was afterwards loaded into Seurat for further analysis.

#### Single-cell sequencing data analysis

Additional quality control as well as sample integration, cell clustering and marker gene identification were done with R (v3.4.1) [39] using Seurat packages v2.3.0 (quality control, normalization) [40] and v3.0.0 (scaling, integration, clustering, differential expression) [41]. Genes that were detected in ≥3 cells and cells in which the number of detected genes ranged between 500 and 6000 (hypoxia samples) or 7500 (normoxia samples) respectively were retained for further analysis. In addition, cells that contained >20% mitochondrial genes were removed, resulting in a total number of 28,114 cells, expressing altogether 20,976 genes. After integration and clustering, another 2264 cells that were likely to be doublets upon examination of marker genes and/or predicted to be doublets by DoubletFinder [42] were excluded. This led to the final dataset containing 25,850 cells (normoxia: *n* = 12,341, hypoxia: *n* = 13,509) and 20,976 genes. This dataset was used for further analysis and graphical display. Gene expression levels were normalized via natural-log normalization of gene transcripts divided by total transcripts per cell and scaled by 10,000. Data integration of the 4 samples was done using the 20 first dimensions of CCA (canonical correlation analysis) and by calling the functions FindIntegrationAnchors() and IntegrateData(). After scaling of integrated data and dimension reduction through principal component analysis, uniform manifold approximation and projection (UMAP) dimension reduction was performed on the first 30 principal components. Subsequently, nearest neighbors were identified using the FindNeighbors() function and clusters were determined with FindClusters (resolution = 0.2). This initially revealed 21 cell clusters. After removal of doublet (sub)clusters (determined by co-expression of 2 main cell-type markers) and merging of clusters that were highly similar with regard to main marker expression, 16 different cell types were identified. Identification of cell types was based on differentially expressed genes (function FindAllMarkers(logfc.threshold = 0.5)) in each cluster and expression of known marker genes [43]. The FindMarkers() function was employed to determine differential expression of *Vegfa* and *Hk2* between hypoxia and normoxia. A positive logFC indicates higher expression under hypoxia as compared to normoxia. Percentages of ODD, tdTomato or double-positive cells as well as *Hk2*+ cells were determined by calculating the proportion of cells with non-zero expression in the respective genes. For all feature plots showing normalized expression levels, a minimal and maximal cut-off was set at 0.5 and 2, respectively.

### Extraction of mRNA and real-time quantitative reverse transcription polymerase chain reaction (qPCR)

Juvenile female WT (C57BL/6N) P32 mice were sacrificed after 6 h of normoxia, hypoxia or CRW (during their active phase, i.e., lights-off) and hippocampi were dissected and directly frozen on dry ice. Total RNA was extracted from hippocampal tissue by using miRNeasy Mini Kit (Qiagen, Hilden, Germany). The cDNA was prepared using SuperScript^®^ III Reverse Transcriptase (Thermo Fisher Scientific Life Technologies GmbH, Darmstadt, Germany), and 1 µg of RNA along with oligo (dT) and Random Hexamer Primer in a total volume of 20 µl. The qPCR was performed as described in detail earlier [31, 44]. For qPCR, 4 µl of 1:10 diluted cDNA were used as template with 5 µl of Power SYBR Green PCR Master Mix (Thermo Fisher Scientific Life Technologies) and 1pmol of primers (in 1 µl H_2_O).

For N-myc downstream-regulated gene 1 protein (*Ndrg1*), nitric oxide synthase 1 (*Nos1*), Ankyrin repeat domain-containing protein 37 (*Ankrd37*), HIG1 domain family member 1A (*Higd1a*), VEGF A (*Vegfa*), erythropoietin (*Epo*), enolase 2 (*Eno2*), *beta*-actin (*Actß*) and hypoxanthine guanine phosphoribosyl transferase (*Hprt1*) the following primers were used:

*Ndrg1* forward primer:

5′-CGAAGACCACCCTGCTCAAG-3′

*Ndrg1* reverse primer:

5′-ATGCTGGCAGAAGGCATGTAT-3′

*Nos1* forward primer: 5′-CATCAGGCACCCCAAGTT-3′

*Nos1* reverse primer:

5′-CAGCAGCATGTTGGACACA-3′

*Ankrd37* forward primer:

5′-AAACAGGTGCTGACCTCAACC-3′

*Ankrd37* reverse primer:

5′-CAGTCCAGGCTTCCAACCTTT-3′

*Higd1a* forward primer:

5′-ACGATGAAGGTCAGGGGTCT-3′

*Higd1a* reverse primer:

5′-AGGCAACAATCGCTGCAAAG-3′

*Vegfa* forward primer:

5′-AGCACAGCAGATGTGAATGC-3′

*Vegfa* reverse primer:

5′-TTGACCCTTTCCCTTTCCTC-3′

*Epo* forward primer:

5′-AAGGTCCCAGACTGAGTGAAAATATTAC-3′

*Epo* reverse primer: 5′-GGACAGGCCTTGCCAAACT -3′

*Eno2* forward primer:

5′-TGGAGTTTGGGGAGTGCTGGATG-3′

*Eno2* reverse primer:

5′-AGGGCTGGGGAGAGGGTTAGAGG-3′

*ß-actin* forward primer:

5′-CTTCCTCCCTGGAGAAGAGC-3′

*ß-actin* reverse primer:

5′-ATGCCACAGGATTCCATACC-3′

*Hprt1* forward primer:

5′-GCTTGCTGGTGAAAAGGACCTCTCGAAG-3′

*Hprt1* reverse primer:

5′-CCCTGAAGTACTCATTATAGTCAAGGGCAT-3′

The qPCR reactions (3 technical replicates) were run on LightCycler^®^ 480 System (Roche, Mannheim, Germany). Fold difference in mRNA expression was calculated by the ΔΔCt method and normalized to *Hprt1* and *ß*-actin.

### Statistical analyses

Data obtained for all quantifications were analyzed by GraphPad Prism 7 (GraphPad Software, Inc. San Diego, CA, USA). Statistical significance between multiple groups was calculated by 1-way or 2-way analysis of variance (ANOVA). Welch’s *t*-test was performed to compare two groups. Jonckheere-Terpstra trend test was applied to identify stepwise increases in data. General and cell-type-specific differences in numbers of *Hk2*+ cells were assessed via Chi-square tests (unadjusted *P* values reported). Differential expression (scRNA-seq) was evaluated by Wilcoxon rank sum test (Bonferroni-corrected *P* values reported, if not declared otherwise). A *P* value < 0.05 was considered statistically significant. Bar graphs show means and error bars represent standard error of mean (SEM). Sample sizes were selected based on previous work in order to allow sufficient statistical power to detect differences with a minimum number of animals (RRR principle). Datasets were routinely screened for statistical outliers using Grubb’s test and outliers were excluded if indicated (*P* < 0.05).

*For intercorrelation analyses and calculation of the gene composite score*, following the reported standard operating procedure [44, 45], gene expression was Z-standardized. Intercorrelation between qPCR-genes was plotted using R-package ‘corrplot’. Cronbach’s alpha was used as measure of internal consistency and *α* > 0.6 considered acceptable. For calculation of the composite score, Z-standardized single gene expression values were averaged across all 7 genes.

## Results and discussion

### Transgenic approach to permanent labelling of hypoxic brain cells

To test our hypothesis that neuronal activity leads to ‘functional hypoxia’, we employed a transgenic mouse line (CAG-CreERT2-ODD::R26R-tdTomato) that allows permanent reporter labelling of cells that undergo hypoxia by expression of a fusion protein. This fusion protein is comprised of the ODD domain of HIF-1α and a tamoxifen-inducible CreERT2, driven by a ubiquitous CAG promoter (slightly modified from Kimura et al. [33]) (Fig. 1a). Using this transgenic approach, we first defined our ‘gold standard’ of a prominent positive control by comparing mice exposed to intermittent inspiratory hypoxia (6%O_2_ for 6 h daily, applied over 5 days) with mice under inspiratory normoxia (21%O_2_) that were handled identically, including the five tamoxifen injections as indicated (Fig. 1b).

### Characterization of the model: hypoxia/tamoxifen and oxygen dose-response curves (inspiratory hypoxia) as well as persistence of labelling

Based on this ‘gold standard’ (tamoxifen injection, followed by 6%O_2_ for 6 h daily, applied over 5 days), we determined the numbers of tdTomato+ cells (mainly neurons—see below) in cornu ammonis hippocampi, namely in CA1, which is a well-defined, highly cognition-relevant, and at the same time established hypoxia-vulnerable brain region (Sommer’s sector) [46–49], and in CA3, as well as in the dentate gyrus (DG) [50, 51]. As deducible from the scheme in Fig. 1b, in pilot experiments, hypoxia days/tamoxifen injections (100 mg/kg/day) had first been experimentally ‘titrated’ by applying 1×, 3× or 5× hypoxia (6%O_2_ for 6 h daily, each preceded by a tamoxifen injection) and compared to respective normoxia days/tamoxifen injections. This led to a clear dose-response of the number of tdTomato+ cells to hypoxia/tamoxifen and our final decision to stay with 5× hypoxia/tamoxifen as our ‘gold standard’ in the following experiments (Fig. 1c, d).

We also explored in initial tests the influence of the degree of inspiratory hypoxia on the number of tdTomato+ neurons and obtained an ‘oxygen dose-response curve’ that started to rise appreciably only at 8%O_2_, and made us select the 6%O_2_ for our subsequent experiments (Fig. 1e, f).

Using pimonidazole as an immediate hypoxia marker for further confirming hypoxia mapping [4] allowed us at least to identify few scattered tdTomato+ cells that showed double-labelling, very similar to what was presented in a recent paper on heart [33]. Unfortunately, the temporal dynamics of tdTomato labelling (visible earliest ~6–8 h after hypoxia exposure) and pimonidazole which can be maximally applied for 90 min before the staining becomes increasingly unspecific, do not permit more extensive co-labelling (Supplementary Fig. 1).

Finally, we analyzed in preparatory work tdTomato labelling at various time points (3 days to 4 weeks) after completed exposure to the standard schedule of 5× tamoxifen injections under inspiratory normoxia (21%O_2_), which showed essentially persistence of labelling over up to 4 weeks (Fig. 1g). In all these experiments, characterizing our transgenic model, 4-week-old male and female mice were equally distributed across experimental groups.

### Light-sheet microscopy discloses a distinct increase in labelled cells from baseline cage activity to inspiratory hypoxia

**Fig. 2:**
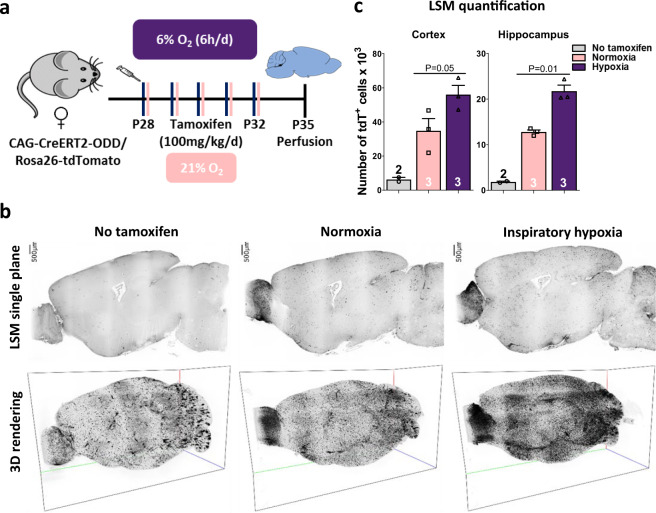
Light-sheet microscopy (LSM) allows spatial mapping of hypoxic cells in whole brain using CAG-CreERT2-ODD::R26R reporter mice. **a** Experimental outline. **b** Light-sheet microscopy (LSM) 2D sagittal planes and maximal intensity projection 3D renderings of tdTomato+ cells in the entire brain hemispheres; images shown in inverted grey scale; scale in 3D images given by bounding box: *x* axis (green) 10 mm, *y* axis (red) 6.2 mm, *z* axis (blue) 4.2 mm. **c** Quantification of tdTomato+ cells in 3D hippocampus and cortex; 2-tailed Welch’s *t*-test; error bars indicate SEM.

We next applied light-sheet microscopy (LSM) to obtain a 3-dimensional (3D) overview of tdTomato+ (ODD) labelling in whole brain (Fig. 2a–c) [37, 52]. Because of the slightly stronger response of female mice to the hypoxia stimulus (see below), we opted for females at the age of 4–5 weeks. Comparative 3D visualization of hypoxic cells revealed a striking, regionally distinct distribution pattern across the entire brain. Similar to our histological quantification (Fig. 1c, d), the amount of labelled cells all over the brain increased markedly from mice with just baseline cage activity to animals exposed to our defined ‘gold standard’ of inspiratory hypoxia (6%O_2_ for 6 h daily, applied over 5 days) (Fig. 2a–c). To control for non-induced expression of the transgene (‘leakiness’ of tdTomato labelling), corn oil (‘no tamoxifen’) was injected, which led to an only small percentage of labelled cells (quantified for normoxia in LSM, Fig. 2c; same depiction upon hypoxia, as screened by histology). We note that the olfactory bulb, the lead sensory organ of mice, showed an extraordinarily strong tdTomato+ labelling upon tamoxifen across conditions, offering in this species an ‘internal control of functional hypoxia’ (Fig. 2a-c and Supplementary Video I).

### Hypoxic cells in cortex and hippocampus are predominantly neurons

**Fig. 3:**
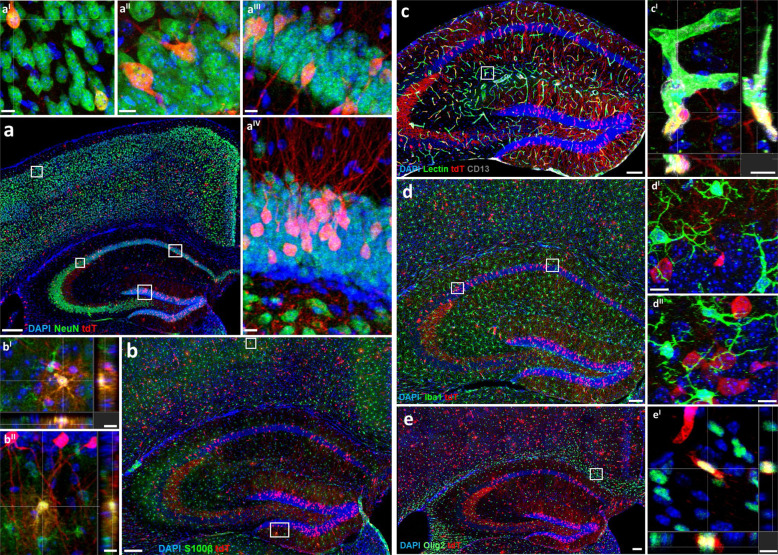
Confocal images illustrate the predominance of neurons among hypoxic cells in hippocampus and cortex of CAG-CreERT2-ODD::R26R-tdTomato mice. Shown are representative images obtained upon our defined ‘gold standard’ of inspiratory hypoxia. *Note that normoxia and CRW (endogenous/activity-related ‘functional hypoxia’) revealed quantitative, no noticeable qualitative differences of labelled cell types*. **a** High density of tdTomato+ (tdT^+^, red) neurons shown by co-labelling with the neuronal marker NeuN^+^ (green). Magnified confocal images of tdT^+^NeuN^+^ neurons depict cortex (a^I^), hippocampal CA2 (a^II^), CA1 (a^III^), and dentate gyrus, DG (a^IV^). **b** S100β-stained astrocytes (green) show less frequent co-immunostaining with tdTomato. (b^I^) and (b^II^) display protoplasmic astrocytes from cortex and DG with orthogonal views. **c** Overview of hippocampal endothelial cells (lectin+, green) and pericytes (CD13+, grey), some co-immunostained with tdTomato; (c^I^) zoom-in image of co-labelled cells. **d** Images of microglia staining (Iba1, green). (d^I^) and (d^II^) represent magnified images from CA1 and CA2, documenting lack of co-localization of Iba1 with tdTomato. **e** Olig2 staining (green) reveals sparse double labelling with tdTomato as illustrated in (e^I^) depicting cells from the corpus callosum. Scale bars represent 200 µm in overview and 10 µm in zoom-in images.

We next explored the immunohistochemical nature of hypoxia signal-positive cells in cortex and hippocampus (representative illustrating images upon our defined ‘gold standard’ of inspiratory hypoxia given in Fig. 3a–e). Comparing normoxia (just baseline cage activity) with exogenous/inspiratory hypoxia (and later with endogenous/activity-related hypoxia; see below) revealed essentially quantitative, no noticeable qualitative differences between conditions. In other words, the cell types labelled under normoxia and exogenous hypoxia (as well as later under CRW performance as endogenous/activity-related hypoxia; see below for quantifications), and their overall distribution were widely corresponding. Remarkably, the overwhelming fraction of tdTomato+ cells were neurons, followed by endothelial cells, whereas labelled astrocytes (mainly protoplasmic), oligodendrocytes and pericytes were much scarcer and displayed some regional differences. Rare, labelled oligodendrocytes, for instance, appeared mainly in white matter areas, whereas no labelled oligodendrocytes were found in the hippocampus. Surprisingly, microglia were never tdTomato+ labelled.

### Single-cell transcriptomes show comparable expression of ODD and tdTomato in hippocampal cell lineages under normoxia or hypoxia

**Fig. 4:**
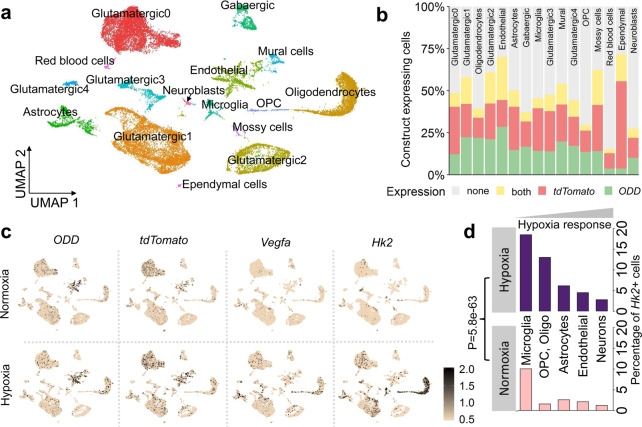
Single-cell transcriptome analysis shows overall construct expression and indicates a role for hexokinase2 in the differential cellular hypoxia response. **a** Unbiased clustering of hippocampal cells from mice under normoxia (*n* = 2) or hypoxia (*n* = 2), represented in UMAP space, reveals 16 distinct cell populations across conditions. OPC: oligodendrocyte precursor cells. **b** ODD and tdTomato mRNAs are present in all cell types at comparable levels; percentage of cells shown where either ODD, tdTomato, both transcripts together, or none were detected (normalized expression >0). **c** Expression plots of ODD, tdTomato, and of 2 hypoxia-regulated genes, *Vegfa* and *Hk2*. Constructs ODD and tdTomato are expressed at similar levels in all cell types. *Hk2*+ cells were ordered by expression level (highly expressing cells plotted on top by setting FeaturePlot(order = T)) to prevent masking of relatively rare *Hk2*+ cells. **d** Percentage of detectable *Hk2* expressing cells increases to a varying degree in all cell types (borderline in endothelial cells under hypoxia). Microglia reveal the highest number of *Hk2* expressing cells under normoxia and a considerable increase under hypoxia; a strong increase under hypoxia is also observed in OPC and oligodendrocytes. For easier visualization, the cluster of ependymal cells, which was located far away from all other clusters, was shifted upwards on the UMAP2 axis (**a**, **c**). Chi-square test presented.

To exclude a merely technical problem as underlying cause of these surprising cellular differences in labelling, we determined whether the ODD-CreERT2 transgene, harbouring a ubiquitously active (CAG) promotor, is indeed equally expressed in all cell types. For this, we employed single-cell transcriptome analysis of whole hippocampus as a most sophisticated and comprehensive approach. This approach confirmed comparable expression of both transgenes (ODD, tdTomato) in all hippocampal cell types under normoxia as well as upon our defined ‘gold standard’ of inspiratory hypoxia (Fig. 4a–c, Supplementary Fig. 2). As an example, we verified *Vegfa* as a prototypical hypoxia-inducible gene, upregulated in many cell types under hypoxia (for bulk comparison normoxia-hypoxia: *P* = 8.96e−22) and particulary highly expressed in OPC and astrocytes (avg_logFC>0.25, *p*_unadj_ < 0.05; Supplementary Fig. 3a, b).

### Hexokinase2 expression is inversely related to the amount of tdTomato+ cells in each cell type

Searching for a first explanation of the apparent ‘hypoxia tolerance’ of microglia (never tdTomato+ labelled in any gender or treatment condition), we made an unexpected discovery. The expression of hexokinase2 (*Hk2*; catalyzing the first step of glycolysis) under hypoxia was found inversely related to the amount of tdTomato+ cells in each cell type (Fig. 4d). Cell types with a high percentage of *Hk2*+ cells under hypoxia showed a lower number of tdTomato+ cells. Accordingly, microglia showed the most prominent expression compared to all other cell types under normoxic and hypoxic conditions (normoxia, *P* = 6.02e−7, logFC = 0.07; hypoxia, *P* = 6.85e−15, logFC = 0.29). Oligodendrocytes also exhibited high *Hk2* expression under hypoxia in the hippocampus. In line with this, no labelled oligodendrocytes appeared within the hippocampus, but predominantly (yet rarely) in white matter tracts (see also below). Hk2 is a glycolytic enzyme, well-recognized from tumour biology to promote cell survival by hypoxia resistance [53, 54]. Under inspiratory hypoxia, increased numbers of *Hk2*+ cells were identified among almost all cell types (*P* < 0.05 compared to the respective cell-type-specific normoxia numbers) (Fig. 4c, d). Even though highly interesting, Hk2 may not be the only explanation for the observed differences between cell types. Notably, however, we did not observe on mRNA level any indicative expression patterns under normoxia or hypoxia of prolyl-hydroxylases, VHL gene or factor inhibiting hypoxia-inducible factor 1 that could potentially explain differences in tdTomato labelling due to different activity of the ODD degradation pathway in different cell types.

Nonetheless, the absence of tdTomato labelling in microglia is a highly intriguing finding. In some agreement with studies on brain tumours [55], we see in our scRNA-seq data that microglia clearly respond with an upregulation of hypoxia-related transcripts, yet it remains unclear why no ODD stabilization takes place. This could be partially explained by the high Hk2 expression, protecting microglia indirectly from a strong decrease in oxygen levels which would be required for ODD stabilization. Alternatively, a different level of oxygen-dependence of microglia (‘set-point’), a divergent activity of the HIF-1α degradation pathway or cell-type-related molecular features preventing ODD-stabilization could be relevant in this context. Possibilities range e.g. from different abundance of prolyl-hydroxylases or VHL protein, deviating availability of iron, ascorbate, tricarboxylic acid cycle intermediates, or levels of radical oxygen species (some only shown in vitro to be relevant) [56]. Moreover, less tight coupling of microglial and vascular oxygen levels (possibly related to distance from capillaries) may affect ODD-stabilization in microglia. All these speculations will have to be experimentally addressed in the future.

### Number of tdTomato+ neurons upon motor-cognitive challenge suggests ‘functional hypoxia’

**Fig. 5:**
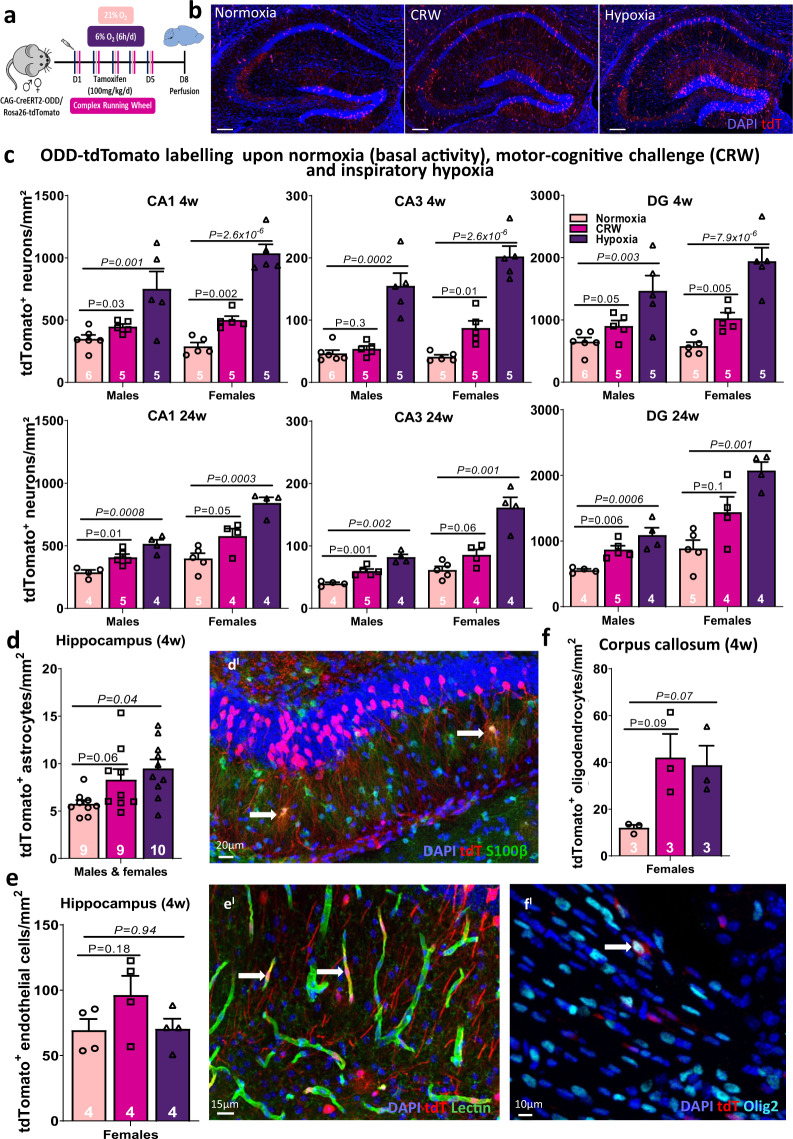
Number of tdTomato+ neurons in hippocampus after motor-cognitive challenge (CRW) versus inspiratory hypoxia support the concept of ‘functional hypoxia’. **a** Experimental outline. **b** Representative images of the hippocampus upon all 3 conditions. **c** Comparative quantification of labelled pyramidal neurons (tdT+NeuN+) in CA1, CA3 and DG of 4 and 24 week-old CAG-CreERT2-ODD::R26R-tdTomato mice of both genders upon spontaneous home cage activity (normoxia), CRW performance and inspiratory hypoxia (‘gold standard’). (**d + d′**) The low numbers of hypoxic astrocytes (tdT+S100β+) in whole hippocampus of both genders display a stair pattern similar to neurons with increase upon CRW and inspiratory hypoxia in 4 week-old mice. (**e + e′**) Quantification of hypoxic endothelial cells (tdT+Lectin+) under normoxia, CRW and hypoxia in whole hippocampus of 4-week-old female mice does not yield significant differences between conditions. (**f + f′**) Quantification of hypoxic oligodendrocytes (tdT+Olig2+) in corpus callosum under normoxia, CRW and hypoxia in 4-week-old female mice shows a strong tendency of increases under both CRW and hypoxia. White arrows in d′, e′ and f′ illustrate quantified double-positive cells; two-tailed Welch’s test for two-group comparison between normoxia and CRW (*P* value non-italicized). Jonckheere–Terpstra trend test (with 20,000 permutations in d–f) for comparing all three groups (*P* value in italics); error bars indicate SEM.

Based on the characterization of our model and its validation by intermittent exogenous/inspiratory hypoxia, we next approached our hypothesis that neuronal activity leads to ‘functional hypoxia’. For this purpose, we selected a multifaceted, demanding learning task for mice, i.e., running on complex wheels [35, 36]. CRW performance requires hippocampal involvement, but also activation of the motor circuits, somatosensory or visual cortex, and recruitment of the prefrontal-corticostriatal path [57–59]. Randomized missing bars, requiring continuous step-length adaptation and bilateral coordination characterize CRW. The CRW task is entirely dependent on voluntary mouse activity and constitutes a marked cognitive challenge that stimulates complicated pattern recognition, intricate motor learning, and respective coordination. We thus compared mice, running on complex wheels, employed to induce our postulated, activity-related, ‘functional endogenous hypoxia’ (‘CRW’; under inspiratory normoxia; 21%O_2_), with mice upon spontaneous cage activity (unchallenged ‘normoxia’ controls; 21%O_2_) and mice exposed to our ‘gold standard’ of a prominent positive control (‘hypoxia’; 6%O_2_ for 6 h daily) - all conditions applied over 5 days. Animals of all groups were handled identically, including tamoxifen injections as indicated (Fig. 5a–c). In this series of experiments, we included also gender and age comparison. Hence, we determined tdTomato+ (ODD labelled) neurons upon normoxia (basal activity), cognitive challenge (CRW) or inspiratory hypoxia (6%O_2_), in males and females separately, as well as in 2 different age groups (4 and 24 weeks, Fig. 5c), focusing again on CA1, CA3 and DG as our regions of interest. Altogether, a characteristic stair pattern from normoxia over CRW to hypoxia became obvious (Jonckheere–Terpstra trend test for both genders and time points significant), with female mice showing slightly higher amounts of tdTomato+ cells (for CA1: interaction treatment × gender: 4 weeks: *P*_interaction_ = 0.048, 24 weeks *P*_interaction_ = 0.018; for CA3: *P*_interaction_ = 0.08 and *P*_interaction_ = 0.0001; for DG: *P*_interaction_ = 0.1 and *P*_interaction_ = 0.09, respectively). Interestingly, this is in line with previous reports on gender differences in response to hypoxia [60–62], reflecting e.g. sex steroidal or oestrous cycle influence, perhaps in combination with a gender-diverse ventilatory response or stress perception during single housing. Of note in this context, even cultured male neurons, being more resistant under normoxia, are more vulnerable under strong hypoxia than female neurons and, interestingly, the male vulnerability pattern is acquired in cells from neonatally testosterone-primed females [63]. Age did not seem to play an appreciable role in the 2 groups (4 and 24 weeks) examined.

To provide a representative impression of the magnitude of the hypoxia response, we quantified in CA1 the percentage of hypoxic, i.e., tdTomato+ neurons among all neurons. These comprised in 4-week-old males 3.70 ± 0.35% upon normoxia, 4.85 ± 0.26% upon CRW and 8.02 ± 1.37% upon hypoxia. In 4-week-old females, fractions amounted to 3.13 ± 0.33%, 5.44 ± 0.46% and 11.2 ± 0.78%, respectively. In 24-week-old males they reached 2.51 ± 0.16%, 4.43 ± 0.35% and 5.66 ± 0.41%, and in 24-week-old females 4.34 ± 0.54%, 6.37 ± 0.75% and 9.28 ± 0.63% (all trend test *P* values <0.01). Importantly, CRW performance as pronounced motor-cognitive challenge, enhanced number of tdTomato+ neurons over normoxia throughout, strengthening our hypothesis that neuronal activity leads to ‘functional hypoxia’, i.e. activity-induced endogenous hypoxia.

Interestingly, also quantification of tdTomato+ astrocytes in whole hippocampus revealed a characteristic stair pattern from normoxia over CRW to hypoxia (Fig. 5d). The percentage of hypoxic astrocytes, i.e. tdTomato+ of all astrocytes, encompassed under normoxia 0.90 ± 0.06%, CRW 1.30 ± 0.17% and hypoxia 1.49 ± 0.14% (trend test *P* = 0.004). In contrast, no significant differences in the number of hypoxic endothelial cells in whole hippocampus were observed between the three conditions (Fig. 5e), with percentages amounting to 7.98 ± 0.91% for normoxia, 9.11 ± 1.34% for CRW and 7.77 ± 0.86% for hypoxia (trend test *P* = 0.61). Overall, we cannot exclude additional region-specific differences (on top of hippocampus), since we did not systematically quantify other brain regions (except for whole cortex in LSM, see below). To provide an extra overview, coronal brain sections illustrate overall brain tdTomato labelling following normoxia versus CRW (Supplementary Fig. 4a, b).

Since in whole hippocampus, there were no hypoxia-labelled tdTomato+ oligodendrocytes found (in perfect accordance with the high *Hk2* expression of these cells, compare Fig. 4d), we had to move to corpus callosum for representative quantifications. There we saw a comparably strong tendency of an increase in hypoxic (tdTomato+) oligodendrocytes upon both, CRW and hypoxia (Fig. 5f). Percentages were as follows: 0.53 ± 0.11% for normoxia, 1.26 ± 0.14% for CRW and 1.91 ± 0.06% for hypoxia (trend test *P* = 0.002). These cell-type-specific findings—in addition to neurons—are highly interesting but will have to be investigated in more depth in future work, employing e.g. different reporter systems and challenges.

We next planned to demonstrate by cFos labelling that induced neuronal activity leads to ‘functional hypoxia’, searching for double-labelling by cFos and tdTomato+ of those neurons that have been activated during CRW. However, the ODD-tdTomato labelling occurs much too late, i.e., when cFos expression has already disappeared; thus, double-labelling cannot be detected due to the different cFos/ODD-tdTomato kinetics [32]. For instance, at 4 h after CRW start, there is plenty of cFos labelling (e.g. cFos+ neurons/mm^2^ in CA1: non-runner controls, 40 ± 20.83, versus CRW, 150 ± 24.36; *P* = 0.01), however, essentially no ODD-tdTomato labelling visible yet.

### Hippocampal mRNA expression of hypoxia-regulated genes after 6 h of CRW supports ‘functional hypoxia’

**Fig. 6:**
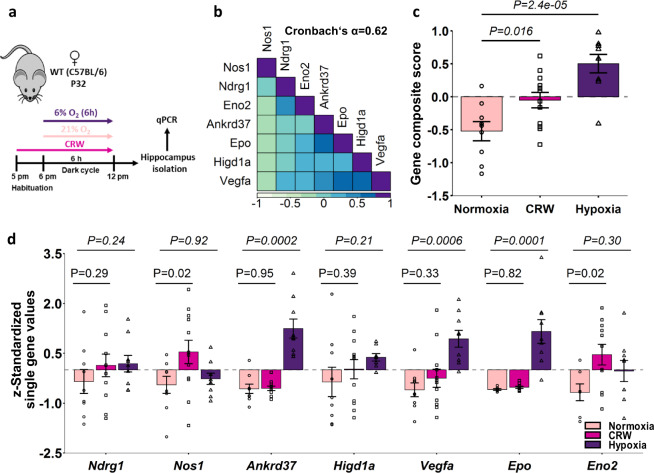
Selected hypoxia-regulated genes’ mRNA expression in hippocampus after 6 h of motor-cognitive challenge versus inspiratory hypoxia support ‘functional hypoxia’. **a** Experimental outline for hippocampal mRNA expression analyses by qPCR. **b** Intercorrelation pattern and Cronbach’s alpha of selected hypoxia-regulated genes. **c** Composite gene score shows clear stepwise increase from normoxia over CRW to hypoxia. **d** Z-transformed qPCR results show comparative expression stair patterns (normalized to *Hprt1* and *ß*-actin) of all selected single genes, namely *Ndrg1, Nos1, Ankrd37, Higd1a, Vegfa, Epo*, and *Eno2*; *t*-test with pooled standard deviation for two-group comparison between normoxia and CRW (*P* value non-italicized); Jonckheere–Terpstra trend test (20,000 permutations in (**d**)) for comparing all three groups (*P* value in italics); error bars indicate SEM.

CRW exposure enhanced the number of tdTomato+ (ODD labelled) neurons over normoxia throughout, supporting that neuronal activity induces ‘functional hypoxia’. Interestingly, intensive treadmill exercise has been reported earlier to increase expression of HIF-1α and its downstream transcript targets [64]. Nevertheless, we next measured hypoxia-regulated gene expression as an additional way of verifying that motor-cognitive challenge causes cellular/neuronal hypoxia. For this, we extracted mRNA from hippocampus of mice, exposed for 6 h in a highly standardized fashion to either normoxia, hypoxia or CRW (Fig. 6a–d). As target genes, we selected *Ndrg1, Nos1, Ankrd37, Higd1a, Vegfa, Epo* and *Eno2*, all confirmed by our scRNA-seq of whole hippocampus or by respective literature [32, 65] to be hypoxia-regulated in neurons and other cell types. We determined mRNA of *Epo* as a very potent hypoxia-inducible gene, which had escaped scRNA-seq analysis due to its very low expression, a known dropout effect of this methodology. For *Epo*, we know from our previous work using in situ hybridization, that CRW induces its expression in pyramidal neurons, however, peaking at 9 h [32].

For most of these selected genes, we see a similar picture as with ODD-tdTomato quantifications, with stepwise increase in hippocampal mRNA expression from normoxia over CRW to hypoxia (Fig. 6d). Although gene expression changes under CRW appear to be slight and are often not nominally significant, the consistently obtained pattern (trend test significant or gene expression CRW/hypoxia>normoxia) at a single defined time point (6 h) is still remarkable, considering variable individual gene expression dynamics and the fact that we exposed mice to only 6 h of CRW, a relatively mild stimulus when compared to 6%O_2_ for 6 h, as used in our positive control. Nevertheless, since we aimed at proving with our selected transcripts and the obtained data that CRW performance indeed causes cellular/neuronal hypoxia, resembling the inspiratory hypoxia configuration, we performed intercorrelation analyses. Based hereon, we calculated a novel composite score of gene expression, following our previously described standard operating procedure [44, 45]. The internal consistency of the mRNA expression data is reflected by a Cronbach’s alpha of >0.6, allowing us to calculate the transcript composite scores for normoxia, CRW and hypoxia, respectively, which turned out to be clearly significant (Fig. 6b–d).

### LSM mapping of tdTomato+ cells upon normoxia versus motor-cognitive challenge indicates ‘functional hypoxia’ in the behaving brain

**Fig. 7:**
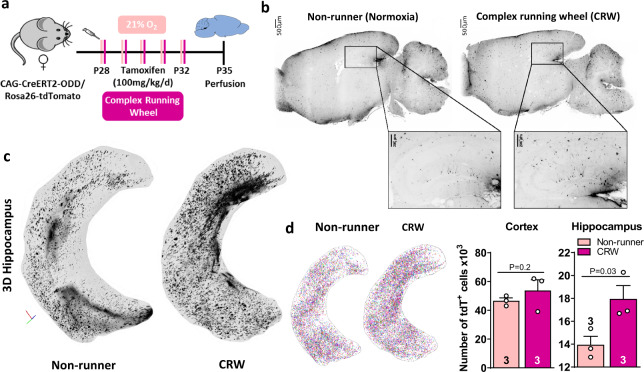
LSM mapping of tdTomato+ cell distribution upon motor-cognitive challenge indicates ‘functional hypoxia’ in the behaving brain. **a** Experimental outline. **b** LSM 2D sagittal planes of tdTomato+ cells; images in inverted grey scale; magnified views of hippocampal area. **c** Dorsal view of 3D hippocampi. Hippocampi cropped from LSM hemisphere dataset and visualized as 3D maximal intensity projection; orientation given in left corner. **d** Quantification of tdTomato+ cells in hippocampus and cortex; left images display 3D renderings shown in (**d**) with tdTomato+ cells represented as random-coloured blobs; 1-tailed Welch’s *t*-test, error bars indicate SEM.

Having substantiated by transcript analyses that motor-cognitive challenge causes endogenous hypoxia, we conducted a second round of LSM analyses. We directly compared female non-runner mice (normoxia) with mice exposed to CRW (Fig. 7a–d). Display in 3D and quantification of tdTomato+ cells in hippocampus revealed a marked increase upon CRW, again strongly supporting our ‘functional hypoxia’ hypothesis (Fig. 7a–d; Supplementary Video II). Strikingly, however, upon performing a particular task (here: CRW) requiring an activation of defined groups of neurons, one might perhaps expect more region-specific activation. The fact that whole hippocampus (and not just *cornu ammonis*) showed an increase in tdTomato+ cells, and that even the cortex displayed a respective tendency, seems less puzzling in light of recent reports that demonstrate a global/brain-wide neuronal activation during certain tasks and behaviours in mice [66] and zebrafish [67]. Moreover, even normal wheel running leads to increased brain activation (measured by cFos expression) in the majority of 25 investigated brain regions [68]. Thus, the focused ‘functional hypoxia’ in hippocampal regions upon CRW that we see is embedded in a global/brain-wide neuronal activation. Of note, some degree of overreporting by the used reporter system, caused e.g. by tamoxifen-induced cellular stress [69], cannot be entirely ruled out. While overall, this does not invalidate the above conclusions, future studies might want to co-administer vitamin E with tamoxifen in order to reduce any potential undesired cellular stress.

### Working model of activity-induced hypoxia as a driver of neuroplasticity

The present data suggest a novel working model in which energy-consuming neuronal activity induces hypoxia with its well-known transcriptional programme that—via HIF stabilization—includes metabolic adaptations and the expression of potent growth factors like EPO and VEGF [10, 13–16]. According to this model, activity-induced physiological hypoxia hereby acts as a critical driver of neuroplasticity, engaging e.g. brain-expressed EPO [32]. Notably, recent work even widened our understanding of the hypoxia response, highlighting hundreds of genes expressed under low oxygen, yet most unrelated to HIF [10, 12]. This suggests that the entire hypoxia response is only in part detectable with our ODD reporter that depends on HIF stabilization. The mechanism is even more complex, as HIF-1α stabilization can also be stimulated e.g. by the PI3K-Akt-mTOR pathway [70], suggesting that HIF-1α dependent gene regulation is kept in a cell-type-specific physiological window. Thus, also CreERT2-ODD dependent activation of the reporter gene may be cell type-specifically co-regulated at the cellular level, partly explaining response differences between cell types, as discussed above and exemplified particularly by microglia that appear nearly unresponsive to hypoxia.

Therefore, all data presented in this manuscript have to be understood in this greater context. We are also aware that functional hypoxia due to specific neuronal network activity, as induced here by CRW, might with our reporter system not equally be detectable in all theoretically involved brain regions (hippocampus versus motor cortex), since HIF may not be the exclusive mediator of the entire hypoxia response. In fact, we see with our reporter a brain-wide ‘general’ neuronal hypoxia answer to tasks such as complex motor-cognitive learning, on top of stimulated specific areas like the hippocampal cornu ammonis (demonstrated in Video II). This response includes the participation of indirectly activated neurons and of non-neuronal cells in adjusting the brain to specific activity-induced challenges. CRW being a strong motor-cognitive test, we do expect both components (general and specific) to be involved. In addition, ‘systemic reactions’ *(‘out of breath’)*, evoked by physical exercise, may add globally to this particular hypoxia response. Future experiments will further tackle these thought-provoking, still open questions and help disentangle the relative contributions of general versus specific components.

To conclude, our ‘functional hypoxia model‘ integrates not only the specific activity-induced hypoxia, as described here for the hippocampus, but may also help understanding previously unexplained observations. These range from the ‘hypoxia vulnerability’ of brain areas highly relevant for cognition, as the CA1 region of the hippocampus (Sommer’s Sector) [46–49], or the observed HIF-1α expression upon exercise in activity-involved brain areas [64], to the beneficial effects of HIF stabilizers on cognitive performance [16]. Moreover, our model suggests a general response to specific activity-induced neuronal challenges, such as complex motor-cognitive tasks, which includes indirectly activated neurons and non-neuronal cells. The widespread ‘functional hypoxia’ arising from increasing energy demands may explain the advantageous effects of physical and cognitive challenges on brain dimensions and global brain function. A picture of hypoxia-induced adaptive neuroplasticity in the postnatal/adult brain is emerging that could also provide the ground for exploring the beneficial therapeutic role of hypoxia in pathological states.

## Supplementary information

Supplementary Figures

Supplementary Video I

Supplementary Video II

## Data Availability

Raw and processed scRNA-seq data are publicly available on GEO via accession code GSE162079.

## Code availability

Analysis scripts for scRNA-seq analysis are available at https://github.com/AgnesSteixner/Butt_et_al_ODD_hypoxia
