# Supplementary material for: Hippocampal neurons respond to brain activity with functional hypoxia
Source: Mol Psychiatry. 2021 Feb 9;26(6):1790–807. doi: 10.1038/s41380-020-00988-w (PMC8440186; doi:10.1038/s41380-020-00988-w)
Supplement: Supplementary file 1 — Supplementary Figures [file 41380_2020_988_MOESM1_ESM.pdf]

SUPPLEMENTARY INFORMATION

Hippocampal neurons respond to brain activity with functional hypoxia

Umer Javed Butt[1§], Agnes A. Steixner-Kumar[1§], Constanze Depp[2§], Ting Sun[2,3],
Imam Hassouna[1], Liane Wüstefeld[1], Sahab Arinrad[1], Matthias R. Zillmann[1], Nadine Schopf[1], Laura Fernandez Garcia-Agudo[1],
Leonie Mohrmann[1], Ulli Bode[2], Anja Ronnenberg[1], Martin Hindermann[1], Sandra Goebbels[2], Stefan Bonn[3], Dörthe M. Katschinski[4],
Kamilla W. Miskowiak[5], Klaus-Armin Nave[2]*, and Hannelore Ehrenreich[1]*

[§]*Shared first authorship*

[1]Clinical Neuroscience and [2]Department of Neurogenetics, Max Planck Institute of Experimental Medicine, Göttingen, Germany
[3]Institute of Medical Systems Biology, Center for Molecular Neurobiology,
University Clinic Hamburg-Eppendorf, Hamburg, Germany
[4]Institute for Cardiovascular Physiology, University Medical Center Göttingen,
Georg-August-University, Göttingen, Germany
[5]Psychiatric Centre Copenhagen, University Hospital, Rigshospitalet, Copenhagen, Denmark

***Correspondence:**
Prof. Hannelore Ehrenreich, MD, DVM
Clinical Neuroscience, Max Planck Institute of Experimental Medicine
Hermann-Rein-Str.3, 37075 Göttingen, GERMANY
Phone +49-551-3899615; Fax +49-551-3899670
E-Mail: **ehrenreich@em.mpg.de**

Prof. Klaus-Armin Nave, PhD
Neurogenetics, Max Planck Institute of Experimental Medicine
Hermann-Rein-Str.3, 37075 Göttingen, GERMANY
Phone: +49-551-3899754; Fax: +49-551-3899758
E-Mail: **nave@em.mpg.de**

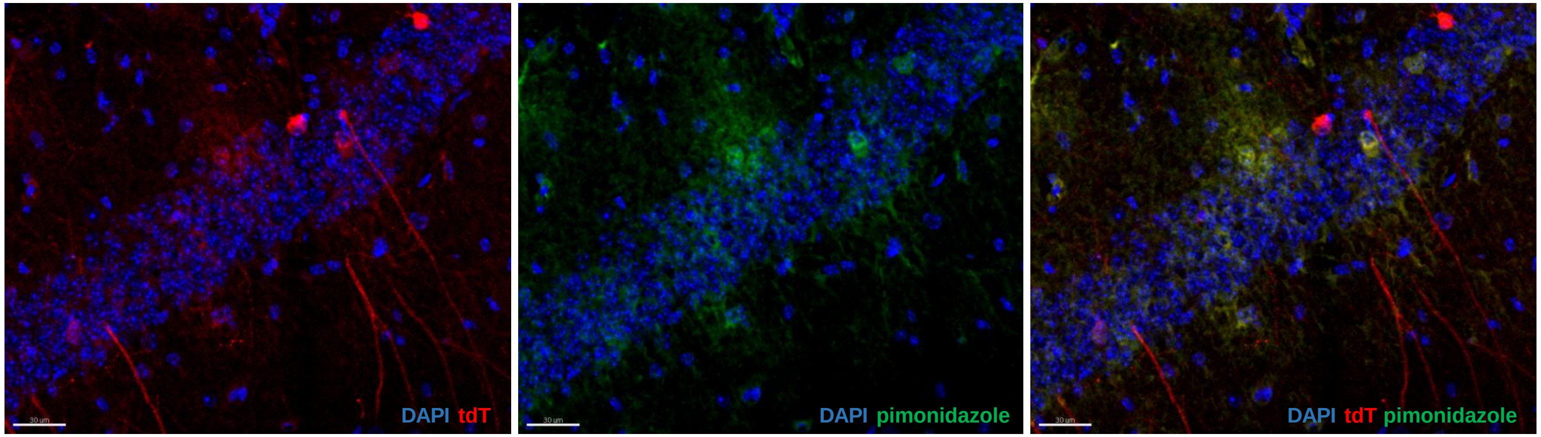

Supplementary Figure 1: Pimonidazole staining confirms functional hypoxia in tdTomato+ cells. Co-labelling of tdTomato and pimonidazole (*Sato et al 2011*) shows the presence of hypoxia in a subset of tdTomato+ cells (compare *Kimura et al 2015*). Pimonidazole was administered 16 hours after 1X tamoxifen injection (100mg/kg i.p.) and CRW start, and mice were sacrificed 90 minutes after pimonidazole administration. Scale bar represents 30 µm.

References:

Sato, Y., Endo, H., Okuyama, H., Takeda, T., Iwahashi, H., Imagawa, A., et al. Cellular hypoxia of pancreatic β-cells due to high levels of oxygen consumption for insulin secretion in vitro. *Journal of Biological Chemistry* **286**, 12524-12532 (2011).

Kimura W, Xiao F, Canseco DC, Muralidhar S, Thet S, Zhang HM et al. Hypoxia fate mapping identifies cycling cardiomyocytes in the adult heart. *Nature* 2015; **523**(7559): 226-230.

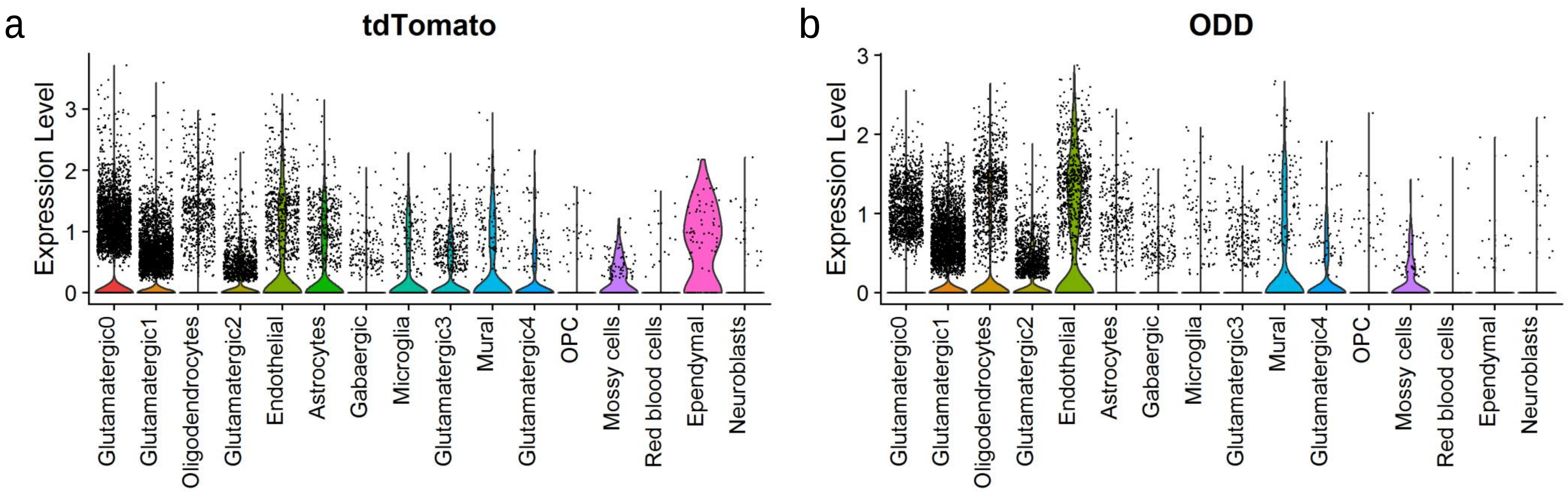

Supplementary Figure 2: Normalized expression levels of (a) tdTomato and (b) ODD in respective cell clusters. No differences in mean expression of either tdTomato or ODD ($p>0.2$, Wilcoxon test, 2-tailed) were observed between microglia and neurons, i.e. the least (microglia) and the most pronounced (neurons, comprising glutamatergic0-4, gabaergic and mossy cell clusters) hypoxia-labelled cell types.

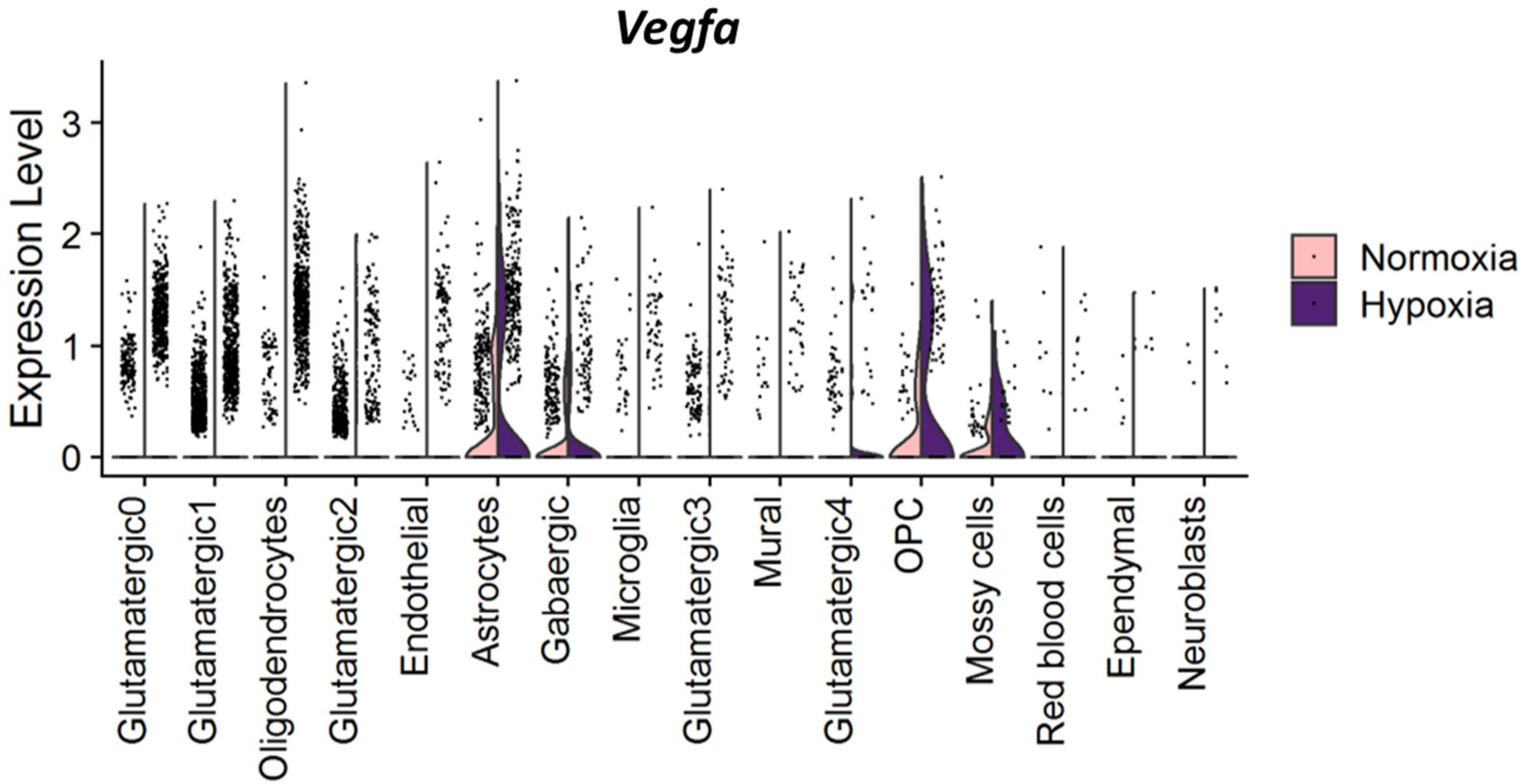

Supplementary Figure 3a: Violin plots showing expression of *Vegfa* under normoxia and hypoxia in all hippocampal cell populations. *Vegfa* was highest expressed in OPC and astrocytes (avg_logFC>0.25, $p_{unadj}<0.05$) when compared to all other cell types.

Butt et al Supplementary Figure 3b

Cluster	p_val	avg_logFC	Hypoxia proportion	Normoxia proportion	p_val_adj
Glutamatergic0	1.06E-32	0.221406	0.118	0.044	2.0E-28
Glutamatergic1	6.28E-04	0.169033	0.173	0.163	1.0E+00
Oligodendrocytes	4.84E-20	0.468132	0.228	0.092	9.2E-16
Glutamatergic2	1.27E-01	0.205379	0.222	0.225	1.0E+00
Endothelial	3.62E-03	0.211827	0.101	0.057	1.0E+00
Astrocytes	1.68E-05	0.470057	0.374	0.35	3.2E-01
Gabaergic	8.91E-01	0.154432	0.258	0.293	1.0E+00
Microglia	6.40E-01	0.09817	0.153	0.188	1.0E+00
Glutamatergic3	3.30E-03	0.313916	0.225	0.171	1.0E+00
Mural cells	2.00E-01	0.049427	0.129	0.203	1.0E+00
Glutamatergic4	4.94E-01	0.317821	0.25	0.246	1.0E+00
OPC	6.77E-03	0.531906	0.389	0.293	1.0E+00
Mossy cells	4.25E-02	0.141386	0.417	0.293	1.0E+00
Red blood cells	6.89E-01	-0.05511	0.108	0.129	1.0E+00
Ependymal cells	1.92E-01	0.255091	0.174	0.086	1.0E+00
Neuroblasts	4.49E-01	0.141355	0.116	0.075	1.0E+00

Supplementary Figure 3b: Differential expression testing for *Vegfa* (as shown in Figure 4a) under hypoxia versus normoxia in all cell types (Wilcoxon test, 2-tailed); **avg_logFC**: positive log fold-change values indicate upregulation under hypoxia, negative values downregulation under hypoxia. **Hypoxia proportion**: Proportion of cells expressing *Vegfa* under hypoxia; **Normoxia proportion**: Proportion of cells expressing *Vegfa* under normoxia. P_val_adj: Bonferroni adjusted p-value (adjusted for all genes in data set).

Butt et al Supplementary Figure 4a

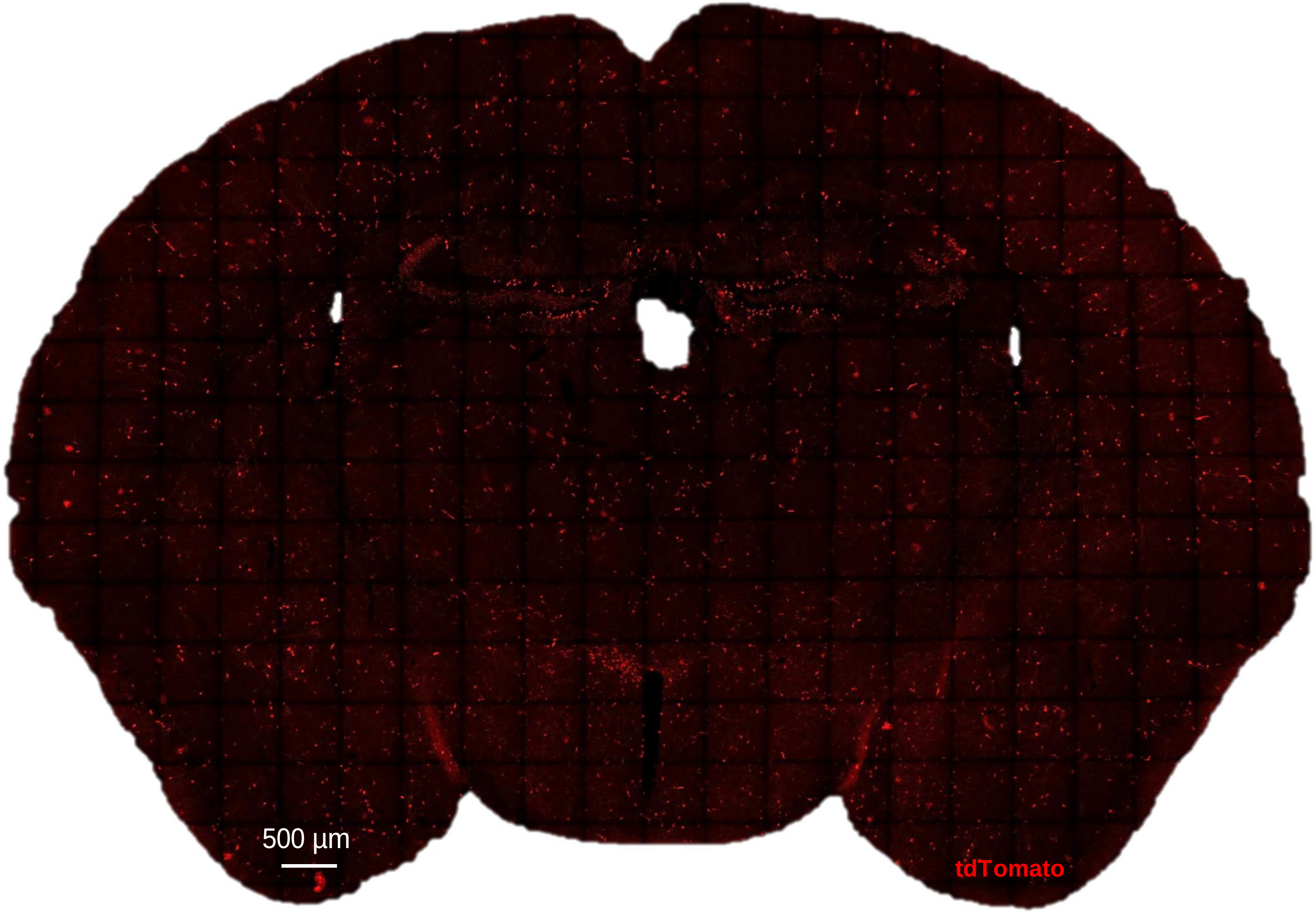

Supplementary Figure 4a: Representative coronal brain section of a CAG-CreERT2-ODD::R26R-tdTomato mouse shows brain-wide distribution of scattered tdTomato+ cells under normoxia (5X tamoxifen injections).

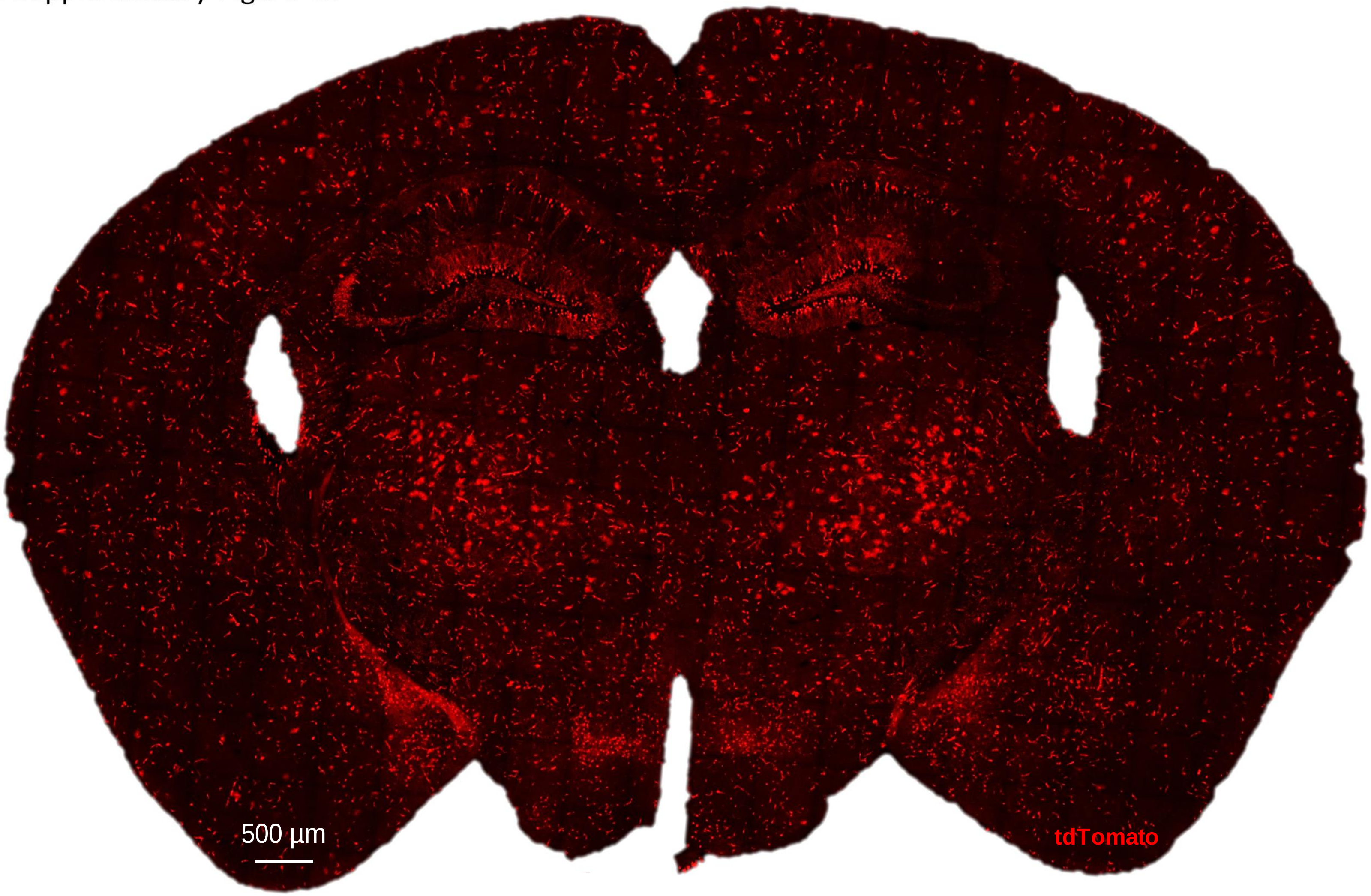

Supplementary Figure 4b: Representative coronal brain section of a CAG-CreERT2-ODD::R26R-tdTomato mouse shows globally enhanced numbers and intensity of tdTomato+ cells after exposure to CRW (5 consecutive nights, 5X tamoxifen injections). The widely distributed tdTomato labelling indicates brain-wide response to motor-cognitive challenge.
